# Clinical characteristics of nummular headache and differentiation between spontaneous and posttraumatic variant: an observational study

**DOI:** 10.1186/s10194-019-0981-4

**Published:** 2019-04-08

**Authors:** Javier Trigo, David García-Azorín, Enrique Martinez Pias, Álvaro Sierra, Alba Chavarría, Angel Luis Guerrero

**Affiliations:** 1 Headache Unit, Neurology Department, Hospital Clínico Universitario de Valladolid, Avda. Ramón y Cajal 3, 47005 Valladolid, Spain; 2 Institute for Biomedical Research of Salamanca (IBSAL), Salamanca, Spain; 3 Department of Medicine, University of Valladolid, Valladolid, Spain

**Keywords:** Allodynia, Cranial trauma, Nummular headache, Precipitants

## Abstract

**Background:**

Head trauma has been described as a precipitating event in Nummular Headache (NH). We aimed to describe the largest NH published series and compare characteristics between idiopathic and post-traumatic cases.

**Methods:**

Patients attended in a Headache Unit in a tertiary hospital (January 2008–January 2018). NH diagnosed according to International Classification of Headache Disorders (ICHD) criteria. We prospectively considered clinical and epidemiological data, comparing idiopathic cases with those precipitated by a cranial trauma.

**Results:**

We included 225 patients (145 women, 80 men) with NH. Median latency between onset and diagnosis was 10 months (IQR: 5–24). Symptomatic treatment was used in 190 patients (84.4%) among which 142 (74.7%) experienced response to it. Preventive treatment was necessary in 127 patients (51.4%), among which 95 (74.8%) achieved response. 29 patients (23 women, 6 men) described a head trauma related to beginning of pain. When comparing groups with or without previous trauma, age of onset was higher among post-traumatic patients (59.9 ± 17.4 vs 48.1 ± 18 years, p: 0.001). Allodynia upon palpation was encountered more frequently in trauma triggered painful areas (53.3% vs. 32.7%, p: 0.02). No other clinical characteristics differences were observed.

**Conclusion:**

Cranial trauma is not a rare trigger of NH. Patients with post-traumatic forms are older and the presence of allodynia is more frequent.

## Background

Nummular headache (NH) was firstly described in 2002 by Pareja et al. as a headache restricted to a small cranial area which can be round or oval shaped, and with a painful area easily delineated by the patient’s finger, ranging from 1 to 6 cm diameter [1]. Since them, more than 280 cases have been reported worldwide [2] and it has been included in the International Classification of the Headache Disorders (ICHD) since the second edition [3, 4].

NH is slightly more frequent in women (1.8:1) and symptoms usually start in the fourth decade of life. Due to the lack of large-scale epidemiological studies and the fact that some people might not require medical attention, incidence is not currently defined [2]. In the largest cases series published to date, which comprised 72 patients, NH corresponded to the 4.6% of all patients evaluated in a headache clinic during the inclusion period [5].

NH is usually considered to be primary or idiopathic. However, different precipitating events have been reported: 8 cases of NH related with head trauma [1, 6–9], 1 with insect sting [10], 1 with varicela-zoster infection [11] and 2 with cranial surgery [12, 13], when an event occurred in close temporal and spatial relationship with the NH onset. There are also secondary cases described: 16 cases have been associated with autoimmune diseases [14], 5 related with intracranial lesions [13–17], 3 with vascular local processes such as subcutaneous aneurysms or calcic hematoma of the scalp [18, 19], and 1 with craniosynostosis [20], when spatial correlation occurred but temporal relation was not easily established. It is not clear whether idiopathic and precipitated, or primary and secondary NH have the same phenotype.

We aimed to present the largest series of patients to date, considering its clinical particularities, and comparing idiopathic vs posttraumatic NH cases.

## Patients and methods

We carried out an observational descriptive study. The study population included patients attended at an outpatient Headache Clinic in a tertiary centre, which covers a population of 270.000 and receives patients both directly from primary care and from general neurology or other specialities offices. Our inclusion criteria were: 1) Fulfilment of the correspondent ICHD criteria [3, 4] at the moment of diagnosis: continuous or intermittent head pain, sharply contoured, fixed in size and shape, round or elliptical, size from 2 to 6 cm and with not better accounted for by another diagnosis 2) capability to describe precisely the characteristics of the pain. We excluded patients with inconclusive data and those in which the diagnosis could not be confirmed. We also excluded patients with a painful area sized between 1 and 2 cm in diameter (those included in ICHD-III edition but not in ICHD-II edition criteria). The study period was from January 2008 to January 2018. We performed a non-probabilistic sampling, including all consecutive cases.

We collected demographical data such as age at inclusion and at onset of pain, sex, and months of evolution at the moment of diagnosis. We also registered clinical information about headache features, including quality, intensity in a 0–10 visual analogic scale (VAS) (0: no pain to 10: the worst imaginable pain), shape, location, and size considering diameter in centimetres. In patients describing more than one focus of NH, the second was described separately. We assessed temporal pattern considering NH was chronic when there were no significant remission periods, episodic when remission periods longer than 3 months were noticed, and recent when time from onset was less than 12 months. Presence of triggers or exacerbations was also considered.

We defined posttraumatic NH, adapted from the ICHD criteria of headache attributed to traumatic injury of the head and/or neck [4] when: 1) patients described a head concussion trauma within 7 days to the onset of pain, 2) located in the exact same location where the NH appeared, 3) trauma was mild according to ICHD criteria [4].

A complete neurological exam was performed, including local examination evaluating the presence of allodynia, defined as the presence of pain from stimuli which are not normally painful, and touch sensitivity, defined as a different sensitivity of an area comparing with its adjacent area. All patients underwent neuroimaging (either CT scan or MRI), and a full blood test, including immunological screening with acute-phase proteins such as erythrocyte sedimentation rate, and antinuclear antibodies. We gathered symptomatic and preventive use prior to headache unit consultation, preventive treatment started in our unit, and percentage of patients that presented a total spontaneous remission. We considered response as present if a > 50% improvement in monthly days of headache was reached, partial if 30–50% response was obtained and no response if patients improved less than 30%. We assessed doses of most frequently used drugs in detail.

Local ethics committee board approved the study. All patients read and signed a consent form prior to their participation.

We hereby present data by mean and standard deviation in case of normal distribution and median and interquartile range (IQR) if not. We employed SPSS v20.0 for statistical analysis. We used chi-square test for evaluating association of qualitative variables and t-test in the comparison of quantitative. We set significance level at 0.05. We checked that missing data was completely at random and those data were managed by complete-case analysis.

## Results

**Fig. 1:**
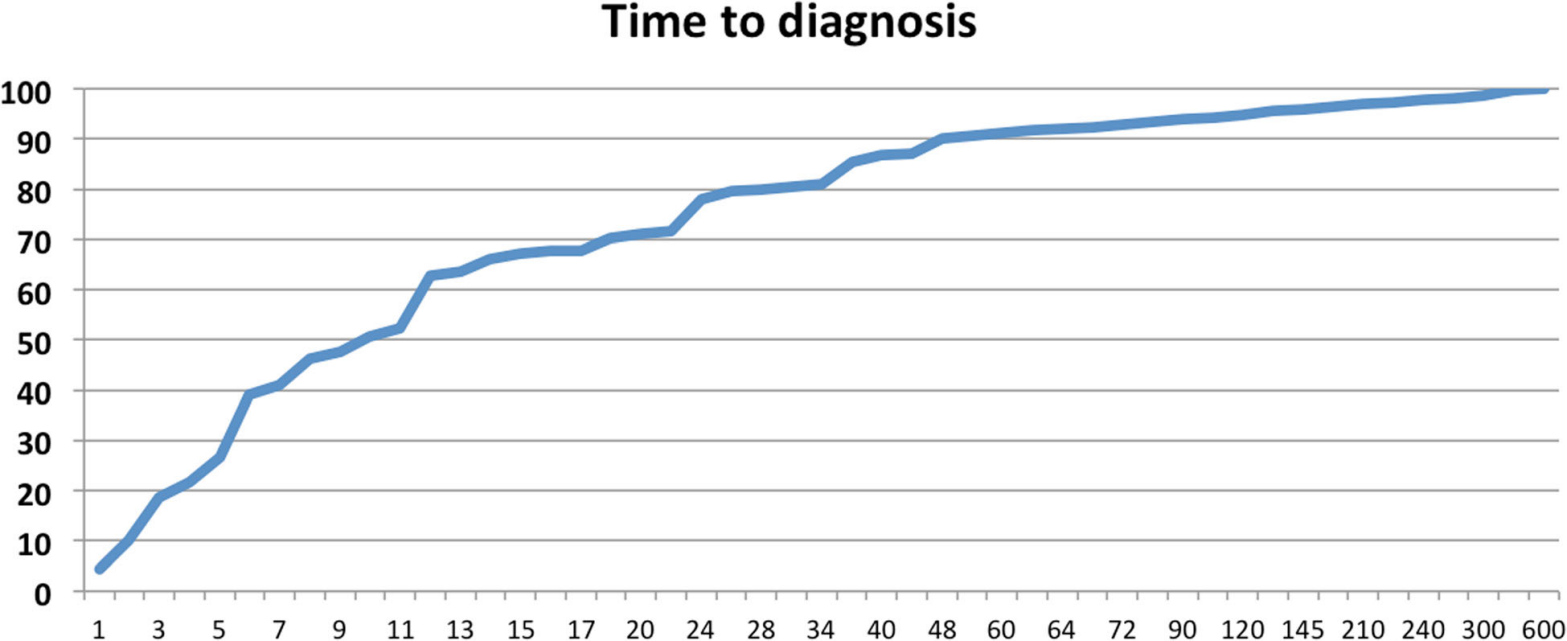
Time to diagnosis. Percentage of patients that have been diagnosed at each given point (y-axis). X-axis represents the time in months to diagnosis

During the study period we evaluated 5515 patients, among which 225 were diagnosed of NH (72 cases were previously published in 2012) [5]. 22 of them (9.7%) described a second NH focus; accounting for a total of 247 pain areas in 225 patients. 145 of the patients were female (64.4%). Mean age at the onset of symptoms was 49.6 +/− 18.3 years. Time of evolution at the moment of diagnosis was 28.4 +/− 63.2 months in mean but 10 [IQR: 5–24] in median. Figure 1 represents the percentage of patients that had been diagnosed until each time period. Temporal pattern was chronic in 98 subjects (43%), recent in 77 cases (34.3%) and episodic in 50 (22.7%) of patients. In episodic patients, mean latency between onset and diagnosis was 52.3 months (106.7), in the chronic group 34.9 months (51.7) and in the group with recent onset 4.7 (2.6) months (p = < 0.001).

### Clinical phenotype

Regarding pain characteristics, background pain was defined as oppressive (42.7%), followed by burning (18.3%), stabbing (17.4%) and throbbing (4.6%), 16.2% of the patients experienced only paroxysmal pain. Mean intensity of basal pain was 5.2 +/1.6 on a visual analogical scale (VAS), and exacerbations were described by 48.6% of the patients with mean intensity of 6.6 +/− 1.3 on VAS. Median duration of exacerbations was 60 s [5–600 IQR]. These exacerbations were described as stabbing in 61.5% of the patients, pressing in 21.4%, burning in 9.4%, tightening in 4.6% and throbbing in 2.6% of the patients (Table 1).

**Table 1:** Demographic and clinical features of NH (n: 225)

Item	Percentage
Gender (M/F)	36.6 /64.4%
Age (years)	52.4 ± 17.9
Age at onset (years)	49.6 +/− 18.3 years
Evolution (months)	28.4 +/− 63.2
Quality	
-Opressive	42.7%
-Burning	18.3%
-Stabbing	17.4%
-Throbbing	4.6%
Side (R / L / S)	50 / 40.1 / 9.9%
Location	
-Tuber Parietal	31.2%
-Occipital	23.5%
-Frontal	19.8%
-Temporal	10.1%
Circular/Ovoid	81 / 19%
Intensity (0-10 scale)	5.2 ± 1.6
Temporal pattern	
Chronic / episodic / recent	43 / 22.7 / 34.3%
Allodynia	35.3%
Touch sensitivity	31.2%

Painful area was described as circular in 81% and oval in 19%, with a mean diameter of 4.4 +/− 1.3 cm. Concerning topography of headache, 50.2% of the areas were right sided, 40.1% left sided and 9.7% were parasagittal located. The precise location was reported as parietal (31.2%), occipital (23.5%), frontal (19.8%), temporal (10.1%), or vertex (3.2%).

All patients had a normal neurological examination except for the presence of allodynia (35.3%) or touch sensitivity (31.2%). Neuroimaging study (CT or MRI) was performed in all patients and alterations were observed in 5 cases (2.3%): small subdural hematoma, pineal cyst and hippocampus cortical dysplasia were found in MRI of three different patients while haemangioma and soft-tissue foreign bodies were found in CT in other two patients.

### Treatment

Symptomatic treatment was used in 190 patients (84.4%). Among them, 61 patients (32.1%) reported complete alleviation with it, 81 patients declared partial response (42.3%) and 48 subjects did not experience any benefit (25.2%). The most frequent were common analgesics, in 179 subjects (72.5%).

At the first visit, 157 of the patients had not received any preventive therapy (69.7%). The most frequently employed drug before referral were neuromodulators such as gabapentin and pregabalin in 38.2% of the patients that received any preventive treatment, followed by amitriptyline in 25% of patients. More than one drug had been tried in 10.3% of patients.

During follow up, 48 patients (19.4%) experienced a spontaneous remission without any treatment. We started oral preventive drugs in 127 patients (51.4%), being the most frequent ones gabapentin, in 107 patients (43.3%), Lamotrigine in 27 patients (10.9%), amitriptyline in 17 (6.9%) and pregabaline in 7 (2.8%). Combined gabapentine and Lamotrigine were used in 14 patients (5.7%) and gabapentine and amitriptyline in 9 patients (3.6%). Other drugs were used in a smaller frequency. A > 50% response was obtained in 86 patients (67.7%), 30–50% response in 9 patients in (7.1%), lack of tolerability in 4 (3.1%) and no response to any oral treatment in 28 patients (22.0%).

### Nummular headache after head trauma

29 patients described head trauma related to NH. When comparing epidemiological characteristics between cases precipitated by head trauma and idiopathic cases, age of onset was significantly higher among posttraumatic patients (59.9 ± 17.4 vs. 48.1 ± 18 years, p: 0.001). No difference in gender distribution was found. Allodynia was found to be more frequent in posttraumatic cases (53.3% vs. 32.7%. p: 0.02) and touch sensitivity was also higher in this group (46% vs. 32.7%. p: 0.04). On the other hand, pain intensity, size, temporal pattern or locations were similar in both groups. Percentage of patients that had received treatment was similar (70% vs. 69.1%, *p* = 0.92) being response rate 46.7% in posttraumatic patients compared with 68.8% in the idiopathic group, although differences were not statistically significant (*p* = 0.14). Percentages of symptomatic treatment use were similar in both groups (84.6 vs. 82.1%, *p* = 0.71). (Table 2).

**Table 2:** Comparison between idiopathic NH and post-traumatic cases

	Idiopathic (*n* = 196)	Post-traumatic (*n* = 29)	p
Sex (M/F)	37.8 / 62.2%	20.7 / 79.3%	NS
Age (years)	50.9 ± 17.6	62.5 ± 16.6	0.01
Age at onset (years)	48.16 ± 18.0	49.53 ± 19.4	< 0.01
Evolution (months)	32.1 ± 61	27.9 ± 63.6	NS
Quality			
-Opressive	46.6%	36.7%	NS
-Burning	16.1%	33.3%	NS
-Stabbing	17.1%	20%	
-Throbbing	5.2%	0%	
Side (R / L / S)	50.2 / 40.1 / 9.7%	50 / 40 / 10%	NS
Location			
-Tuber Parietal	31.8%	26.7%	NS
-Occipital	24.4%	16.7%	NS
-Frontal	20.7%	13,3%	
-Temporal	8,3%	23.3%	
Circular/Ovoid	80,6/ 19,4%	83,3 / 16,7%	NS
Intensity	5.2 ± 1.6	5 ± 1.9	NS
Temporal pattern			
Chronic / episodic / recent	42.9 / 23.1 /34%	43.3 / 20 / 36.7%	NS
Allodynia	32.7%	53.3%	0.02
Touch sensitivity	29%	46.7%	0.04

## Discussion

This cases series involves 225 patients diagnosed of NH accordingly to ICHD-III criteria [4], which represents the largest published cases series to date.

NH incidence is not clearly defined. Some patients might not require medical attention or are not referred to neurologist for evaluation, so it might be an under-recognized syndrome. Worldwide epidemiological studies are necessary to reach global incidence conclusions.

In our sample, demographic data such as sex or age of onset concurs with previous published series [2] observing a female predominance (1:1.8) and a main age of onset at fourth decade. Latency between onset and diagnosis (10 months [IQR: 5–24] in median) does not seem too large, though it still reflects in our opinion the lack of knowledge about this relatively new entity among general practitioners and neurologists.

In contrast to other headaches, NH pain do not have a characteristic or specific quality, however, oppressive pain was the most frequently described in our series being present in almost half of the patients. Paroxysms, which are usually frequent in epicranial headaches, are not uncommon in NH and some of our patients referred them.

Since Pareja et al. described NH in 2002; head trauma has been reported as possible precipitant in multiple series. However no comparison has been made between idiopathic and posttraumatic cases. When comparing both groups, we observed a higher mean age and a higher rate of cases associated with allodynia and touch sensitivity in post-traumatic cases. Other demographic characteristics, clinical features and response to treatment were similar in both groups.

Previous literature showed a higher prevalence of posttraumatic headache in elderly population [21]. This is explained by major medical comorbidity in this group and higher prevalence of drops than general population. So it is not rare that posttraumatic NH cases patients in our series were older than the idiopathic group of patients.

NH is considered to be a peripheral local process, supported by the fact that symptoms and signs are confined to a small area. Some authors have suggested a neuralgic pain attributed to terminal neural branches of different cutaneous nerves, but the existence of painful areas located in the midline in some patients, and the lack of response to local anaesthetic injections counterpoints this hypothesis. Structural abnormalities located within different tissue plains from the inner periosteum outwards might be an explanation [22]. It has been suggested that pain as a consequence of superficial C-fibers dysfunction. Considering an epicranial origin of the pain, tissue plains lesions may be more severe in post-traumatic cases, which could explain the higher rate of allodynia and touch sensitivity on physical exam in these cases.

In patients with severe pain, treatment may be necessary. In our series most patients needed symptomatic treatment while more than half required preventive treatment; reflecting that NH can behave as a disabling headache. Preventive treatment was only initiated before referral to our Unit in 31.3% of cases, showing again the lack of knowledge about this headache among general physicians.

**Fig. 2:**
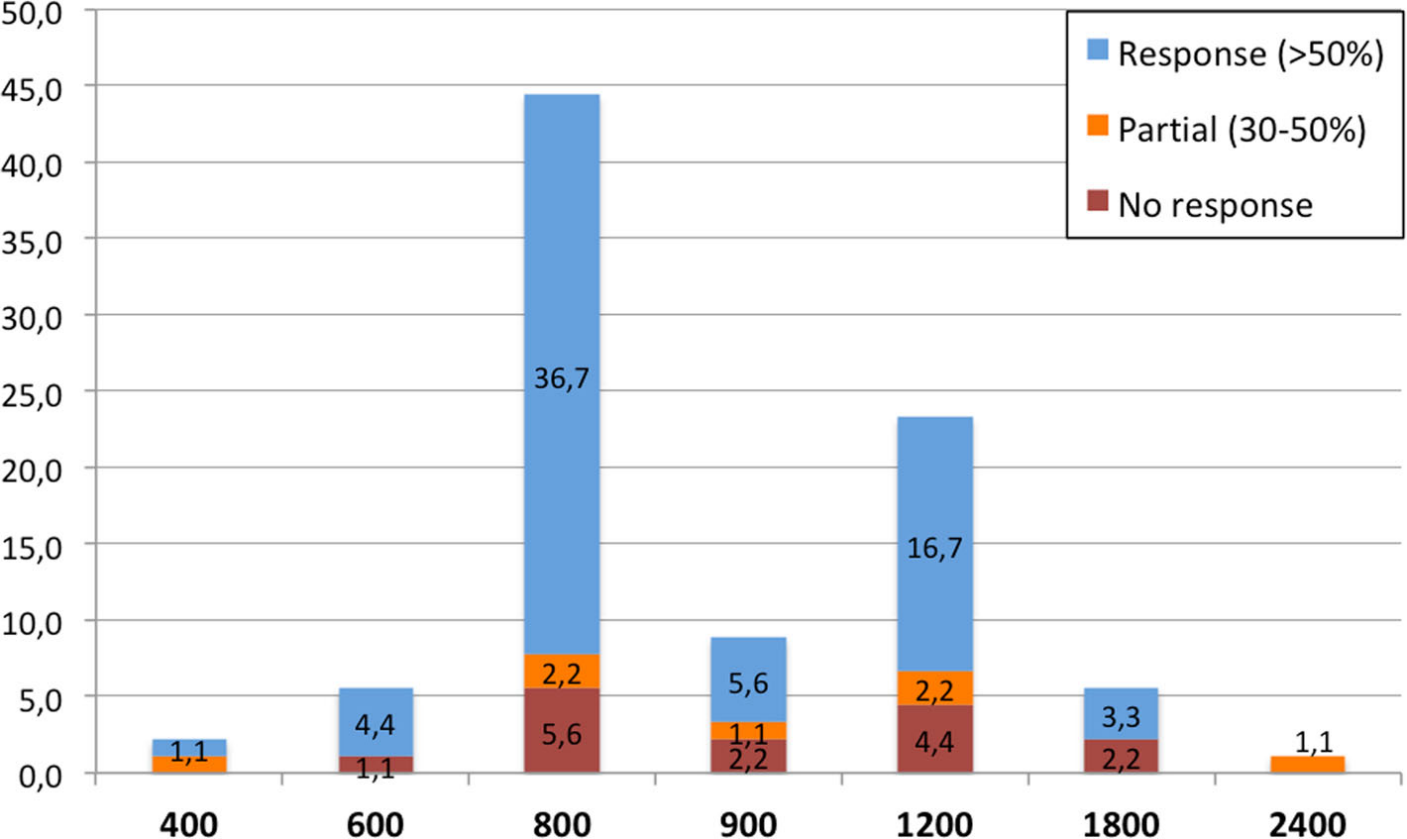
Clinical response to gabapentine. Blue bars: > 50% response, orange bars: 30–50% response, red bars: no response. Percentage represents % of patients treated with gabapentine

There are no clinical trials to base any therapeutic strategy; therefore treatment is usually supported by small case series. Acute pain has been treated with nonsteroidal anti-inflammatory drugs with benefit in 60% of published cases [23]. In our series this percentage was higher, finding benefit (complete or partial) in 74.4% of treated patients. On the other hand, different drugs have been used as preventive therapies and gabapentin have shown to be the most effective treatment among them, with a response rate of 60% [5, 7, 8, 11–13, 24–29]. In our series, three quarters of patients showed some kind of response to preventive treatment. Gabapentin was the most frequently used drug and the most common dose was 800 mg/ daily. We found that response rate, as shown in Fig. 2, seems to be favourable at medium doses, while low doses might not be enough and high doses might not be properly tolerated. Onabotulinum toxin type A may be a reasonable therapeutic approach for those patients refractory to gabapentin or other oral drugs [2].

This study has limitations. Though the patients have been included prospectively in our registry, the antecedent of trauma depends on the memory of patient. We cannot rule out the possibility that some patients did not remember a potentially significant trauma.

In addition, the small percentages of post-traumatic NH patients [29] compared to the primary NH (196) make the statistical interpretation difficult. Therefore, more studies with a bigger size could be necessary to reach solid conclusions.

## Conclusions

Nummular headache is not a rare entity. Although it is usually considered to be a primary headache, secondary cases to different precipitating events have been described, among which head trauma is the most frequent.

We describe the largest published series and we compare primary NH with cases precipitated with head trauma. The group related to head trauma showed a higher rate of allodynia and touch sensitivity. These findings might support an epicranial origin of the pain.

## Abbreviations

ICHD: International Classification of Headache Disorders
NH: Nummular headache
OnabotA: Onabotulinum toxin type A
VAS: analogical scale
